# Structural Connectivity Alterations in Amyotrophic Lateral Sclerosis: A Graph Theory Based Imaging Study

**DOI:** 10.3389/fnins.2019.01044

**Published:** 2019-10-02

**Authors:** Etienne Fortanier, Aude-Marie Grapperon, Arnaud Le Troter, Annie Verschueren, Ben Ridley, Maxime Guye, Shahram Attarian, Jean-Philippe Ranjeva, Wafaa Zaaraoui

**Affiliations:** ^1^Aix Marseille Univ, CNRS, CRMBM, Marseille, France; ^2^APHM, Hôpital de la Timone, Referral Centre for Neuromuscular Diseases and ALS, Marseille, France; ^3^APHM, Hôpital de la Timone, CEMEREM, Marseille, France; ^4^Aix Marseille Univ, INSERM, GMGF, Marseille, France

**Keywords:** ALS, MRI, connectivity, DTI, hub, graph theory

## Abstract

**Background:**

Amyotrophic lateral sclerosis (ALS) is a relentlessly progressive neurodegenerative disorder. Diffusion magnetic resonance imagining (MRI) studies have consistently showed widespread alterations in both motor and non-motor brain regions. However, connectomics and graph theory based approaches have shown inconsistent results. Hub-centered lesion patterns and their impact on local and large-scale brain networks remain to be established. The objective of this work is to characterize topological properties of structural brain connectivity in ALS using an array of local, global and hub-based network metrics.

**Materials and Methods:**

Magnetic resonance imagining data were acquired from 25 patients with ALS and 26 age-matched healthy controls. Structural network graphs were constructed from diffusion tensor MRI. Network-based statistics (NBS) and graph theory metrics were used to compare structural networks without *a priori* regions of interest.

**Results:**

Patients with ALS exhibited global network alterations with decreased global efficiency (Eglob) (*p* = 0.03) and a trend of reduced whole brain mean degree (*p* = 0.05) compared to controls. Six nodes showed significantly decreased mean degree in ALS: left postcentral gyrus, left interparietal and transverse parietal sulcus, left calcarine sulcus, left occipital temporal medial and lingual sulcus, right precentral gyrus and right frontal inferior sulcus (*p* < 0.01). Hub distribution was comparable between the two groups. There was no selective hub vulnerability or topological reorganization centered on these regions as the hub disruption index (κ) was not significant for the relevant metrics (degree, local efficiency and betweenness centrality). Using NBS, we identified an impaired motor subnetwork of 11 nodes and 10 edges centered on the precentral and the paracentral nodes (*p* < 0.01). Significant clinical correlations were identified between degree in the frontal area and the disease progression rate of ALS patients (*p* < 0.01).

**Conclusion:**

Our study provides evidence that alterations of structural connectivity in ALS are primarily driven by node degree and white matter tract degeneration within an extended network around the precentral and the paracentral areas without hub-centered reorganization.

## Introduction

Amyotrophic lateral sclerosis (ALS) is a neurodegenerative motor neuron disorder characterized by progressive upper and lower motor neuron degeneration, leading to severe motor disability and death due to respiratory failure within few years (Kiernan et al., 2011). While the etiology of ALS remains elusive, ALS is now widely regarded as a multisystem disorder with considerable extra-motor involvement (Al-Chalabi et al., 2016; Christidi et al., 2018). Neuroimaging studies in ALS have consistently captured clinico-radiological correlations in the central nervous system (Cirillo et al., 2012; Bede and Hardiman, 2014).

Structural changes are relatively difficult to ascertain in ALS with conventional, clinical magnetic resonance imagining (MRI) sequences, therefore research studies rely on quantitative techniques, such as diffusion tensor imaging (DTI; Grolez et al., 2016), cortical thickness mapping (Schuster et al., 2017; Consonni et al., 2019) or MRI spectroscopy (Kalra, 2019). DTI studies have highlighted fractional anisotropy (FA) reductions in both motor and extra-motor regions (Foerster et al., 2013) and FA proved to be a sensitive DTI metric for both diagnostic (Tang et al., 2015; Bede et al., 2017) and progression modeling (Menke et al., 2012; Müller et al., 2016). Tractography studies in ALS readily detect white matter tract degeneration principally in the corticospinal tracts (Agosta et al., 2010). These studies have described anatomical patterns of white matter degeneration, but the impact of focal white matter changes on brain network integrity has not been fully characterized to date (Bede, 2017).

Structural connectivity studies based on graph theory offer a valuable tool to analyze the topological organization of cerebral networks and elucidate how different brain regions relate to each other (Bullmore and Sporns, 2009). In connectomics, cortical and subcortical brain regions can be parcellated in nodes with white matter tracts between them representing the edges of a mathematical graph. The human connectome has been extensively studied with graph theory in physiological and pathological contexts and it exhibits non-random features such as the presence of highly connected regions, named hubs (Achard, 2006). Connectivity hubs of the human brain support integrative processing and adaptive behaviors with high metabolic demands and represent vulnerable foci of neurodegeneration (Buckner et al., 2009; van den Heuvel et al., 2013; Proudfoot et al., 2019).

In ALS, few structural connectivity studies have reported both motor (Verstraete et al., 2011) and extra-motor node impairment (Verstraete et al., 2014; Buchanan et al., 2015; Dimond et al., 2017) and corticobasal connectivity is seldom evaluated specifically (Bede et al., 2018). However, due to methodological differences, the findings of these studies are inconsistent; some authors have described decreased (Dimond et al., 2017), while others reported preserved global efficiency (Buchanan et al., 2015). While many brain disorders, such as Alzheimer disease (Buckner et al., 2009; Verstraete et al., 2011) or schizophrenia (Rubinov and Bullmore, 2013) exhibit a hub-centered pattern, this finding is not evident in ALS connectivity studies (Crossley et al., 2014). It is still not clear if the clinical manifestations of ALS are primarily driven by white matter degeneration or by hub topology alterations.

Accordingly, the main objective of this prospective MRI study, is the characterization of structural connectivity in ALS using graph theory methods at different scales: global, local (nodal) and network analysis using statistical methods such as the Network-based Statistics (NBS; Zalesky et al., 2010) allowing us to analyze both cortical and white matter integrity.

## Materials and Methods

### Ethics

This prospective imaging study was approved by the regional Ethics Committee (Sud Mediterranee I). All subjects provided informed consent in accordance with the principles of the Declaration of Helsinki.

### Participants

Twenty-five consecutive patients with ALS, diagnosed according to the revised El Escorial criteria (Brooks et al., 2000), were recruited from the ALS Center of Marseille University Hospital, France. Comorbid neurological conditions or coexisting frontotemporal dementia (FTD; Rascovsky et al., 2011) were considered as exclusion criteria. Twenty-six healthy volunteers were also recruited as radiological controls. Healthy controls had no history of prior head injuries, neurological or psychiatric diagnoses and had a normal clinical examination.

### Clinical Evaluation

All ALS patients underwent a standardized clinical examination on the day of the MRI. The recorded demographic and clinical parameters included: disease duration, site of onset, revised ALS functional rating scale (ALSFRS-R) scores (Cedarbaum et al., 1999) and disease progression rate defined as (48-ALSFRS-R)/disease duration (months).

### MRI Acquisition

Magnetic resonance imagining data were acquired on a 3T MAGNETOM Verio system (Siemens, Erlangen, Germany) using a 32-channel phased-array ^1^H head coil. The protocol included a high-resolution ^1^H T1-weighted (T1w) 3D-Magnetization-Prepared Rapid Acquisition Gradient-Echo (MPRAGE) sequence (TE/TR/TI = 3/2300/0.9 ms, 160 slices, resolution of 1 × 1 × 1 mm^3^, acquisition time = 6 min), and a single shot echo-planar imaging DTI sequence (64 encoding directions, *b* = 1000 s/mm^2^ and b0, TE = 95 ms, TR = 10700 ms, slice thickness 2 mm, 60 contiguous slices, resolution of 2 × 2 mm^2^, acquisition time = 12 min). A T2-weighted 3D-Fluid-Attenuated Inversion Recovery (FLAIR) sequence (TE/TR/TI = 395/5000/1800 ms, 160 slices, 1 × 1 × 1 mm^3^ spatial resolution, acquisition time = 6 min) was also performed in both patients and controls and systematically reviewed to ensure the absence of vasculopathic white matter abnormalities which may affect focal diffusivity parameters.

### Image Processing (Figure 1)

#### Anatomical Cortical and Subcortical Parcellation

Cortical and subcortical brain regions of each subject were parcellated using the Freesurfer software^1^ (v5.0) with the Destrieux atlas, based on the T1w MPRAGE images to obtain 164 regions of interest (Destrieux et al., 2010). Subcortical regions included the thalamus, caudate nucleus, putamen, pallidum, hippocampus, amygdala, nucleus accumbens and ventral diencephalon.

**FIGURE 1:**
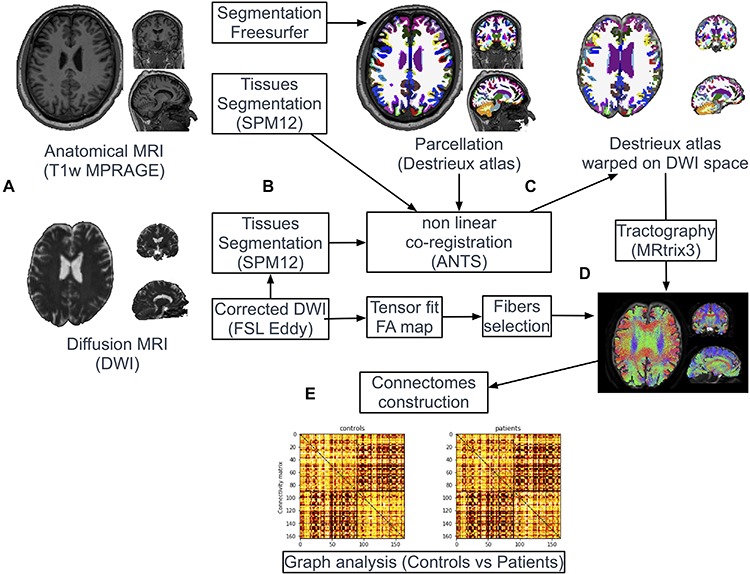
Pipeline of image processing and graph analysis. T1w and diffusion MRI sequences were acquired from all patients and controls **(A)**. Following automatic parcellation of the structural images into cortical and subcortical regions **(B)**, the two set of images were non-linearly co-registered **(C)**. White matter tracts were reconstructed based on diffusion data using the probabilistic algorithm of MRtrix **(D)**. Individual brain network maps were generated using nodes and white matter connections resulting in a FA-weighted connectivity matrix for each subject. Graph analyses were performed on the FA-based connectivity matrix **(E)**.

#### Co-registration Between T1w Images and Diffusion Images

Tissues-type segmentation was performed based on T1w and b0 diffusion images using the Statistical Parameters Mapping software^2^ (SPM12) to extract cerebrospinal fluid (CSF) maps (Figure 1B). T1w and b0 CSF maps were used as source and target images, respectively, to estimate geometric distortions with a non-linear registration procedure (Syn Model of ANTs library (Avants et al., 2008; Figure 1C). The resulting deformation map obtained from this co-registration was applied on the parcellation mask (Destrieux labels). Nearest neighbor interpolation was applied on the mask to keep the integer values of the original labels.

#### Diffusion Image Preprocessing

Each diffusion dataset was aligned to its b0 image using affine registration to correct for head movement and Eddy-currents using FSL^3^ (version 5.0.8, FMRIB Software Library, Destrieux et al., 2010).

#### Tractography and Connectomes Construction

White matter tracts were reconstructed adopting a whole-brain probabilistic fibertracking approach using MRtrix^4^ (Brain Research Institute, Melbourne, Australia). The method has been previously presented in detailed (Besson et al., 2014). A combined bi-hemispheric white matter mask was 1 mm dilated and defined as the region of interest (ROI) for the tracking algorithm (Figure 1D). One million fibers were then generated from all voxels included in this ROI with a probabilistic tracking algorithm [tckgen command, FOD model (Behrens et al., 2003)]. The algorithm generated one million fibers with a minimum length of 20 mm. Default tracking parameters included a minimum radius of curvature of 1 mm, a FOD cutoff of 0.1 and a step size of 0.2 mm. Finally, FA values were interpolated at each point of the fibers and the FA-based connectivity matrix (size 164 × 164) were generated from all streamlines and the parcellation mask (164 labels) previously warped in the diffusion dataset space (tck2connectome command, MRtrix) (Figure 1E).

### Network Construction and Graph Theory Based Analysis

We modeled the structural undirected brain network of each participant using the reconstructed white matter tracts and the parcellated brain regions obtained in the previous steps (Bullmore and Sporns, 2009). Each region was used to define a node of a network graph. Edges were determined by tractography streamlines connecting any pair of nodes. An edge was considered present between two nodes if a streamline was generated with start and end points in each region. Network metrics were computed using the Brain Connectivity Toolbox (Rubinov and Bullmore, 2013). We investigated measures of global network architecture of each subject with global efficiency and mean degree. Local structural alterations were evaluated based on local efficiency, degree and betweenness centrality of each region.

As defined by Rubinov and Sporns (2010), global efficiency (Eglob) is the average inverse shortest path length in a network and is inversely related to the characteristic path length. It is a metric of functional integration that reflects on the brain’s ability for specialized processing across distributed brain areas. Node degree is defined as the number of edges connected to the node and is a fundamental basic network measure to assess the central role of a brain region among a network.

Mean degree of the whole network was also computed and defined for one subject as the mean of each node degree of this subject. Betweenness centrality (BC) is the fraction of all shortest paths in the network that pass-through a given node. The local efficiency (Eloc) is the global efficiency computed on the neighborhood of the nodes.

### Hub Analysis

In connectomics, hubs are described as highly connected nodes with topological centrality and a critical role in integrative processes and adaptive behaviors. Hubs are typically defined as nodes with the highest degree, i.e., a degree one standard deviation higher than the average degree of all nodes in the studied population (van den Heuvel and Sporns, 2011; Llufriu et al., 2017).

To assess if the topologic reorganization was more prevalent in hubs, we also calculated the hub disruption index, κ, as defined by Achard (Achard, 2006). κ index is the gradient of a straight line, plotting the mean value at each node in the healthy controls group (x-axis) versus the difference between patient and control groups at each node, for any given metric (y-axis). For example, a negative κ index, crossing the zero line on the y-axis, reveals a trend for high-value regions in controls to decrease in the studied subject, and low-value nodes to increase.

### Network Based Statistics

Considering a 164-node network, 26732 network connections can theoretically be generated. As a result, standard statistical tests for nodal analyses may be underpowered after correcting for multiple comparisons. Accordingly, we used the NBS approach to identify impaired subnetworks in patients compared to controls. The NBS methodology improves the statistical power by controlling for type I error (Zalesky et al., 2010). The NBS network was computed using the parameters detailed in a previous ALS study (Verstraete et al., 2011) permitting the identification of an altered network derived from the FA-based connectome. The mean FA of each tract of this isolated subnetwork was calculated for each subject and the total FA sum (NBS FA sum) was compared between the two groups.

### Statistical Analyses

Statistical analyses were performed using JMP 9.0.1, SAS Institute Inc (JMP^®^, Version 9. SAS Institute Inc., Cary, NC, 1989–2019). Group characteristics were compared using the non-parametric Kruskal-Wallis test for age and Chi-squared test for gender and handedness. Differences in global connectivity between patient and control groups were assessed using pairwise non-parametric Wilcoxon signed-rank tests for multiple comparisons with uncorrected *p* < 0.05 being considered statistically significant. For each node, group differences in the mean degree, Eloc, and Eglob were also explored with non-parametric Wilcoxon tests (*p* < 0.05).

At the nodal scale, we used two thresholds to study differences between patients and controls: a first *p* = 0.05/164 = 0.0003 threshold corresponding to Bonferroni corrections applied with 164 brain regions, and a p exploratory threshold 1/n (*p* < 0.006) (Ridley et al., 2015).

The connectivity metrics with significant differences between patients and controls were then correlated with clinical parameters including ALSFRS-R score and disease progression rate, using a Spearman Rank test with multiple corrections. A *p* < 0.0125 for global metrics (Eglob and mean degree) and a *p* < 0.004 for local metrics (6 nodes with significant connectivity differences between patients and controls) were considered statistically significant.

## Results

### Demographic and Clinical Parameters

The demographic and clinical profile of the participants is presented in Table 1. Twenty-five patients with ALS (mean age 55; SD 10 years; 16 males and 9 females, 2 left-handed) and 26 healthy controls (mean age 51; SD 10 years; 15 males and 11 females, 5 left-handed) were included. There was no statistical difference in age (*p* = 0.17), gender (*p* = 0.64) and handedness (*p* = 0.24) between the study groups. The mean ALSFRS-R score in the ALS cohort was 39 ± 6.

**TABLE 1:** Demographic and clinical parameters of ALS patients and controls.

**Demographic and clinical variables**		**ALS patients**	**Healthy controls**	***P*-value**
*n* (male/female)		25 (16/9)	26 (15/11)	0.64
Age mean ± SD (years)		55 ± 10	51 ± 10	0.17
Handedness (right/left)		23/2	21/5	0.24
Disease duration mean ± SD; [range] (months)		18 ± 15; [5–61]	N/A	N/A
Disease onset site	Spinal	*n* = 19 (left LL: *n* = 6, right LL: *n* = 7, left UL: *n* = 1, right UL: *n* = 5)	N/A	N/A
	Bulbar	*n* = 6	N/A	N/A
Revised El-Escorial criteria	Definite	*n* = 5	N/A	N/A
	Probable	*n* = 10	N/A	N/A
	Probable Laboratory Supported	*n* = 5	N/A	N/A
	Possible	*n* = 5	N/A	N/A
ALSFRS-R mean ± SD; [range]		39 ± 6; [23–47]	N/A	N/A
Disease progression rate mean ± SD; [range]		0.9 ± 0.9; [0.1–3.6]	N/A	N/A

*ALSFRS-R, revised ALS functional rating scale; LL, lower limb; N/A, not applicable; UL, upper limb.*

### Global Metrics

Global network analyses are presented in Table 2. Compared to controls, ALS patients showed a significant decrease in Eglob (0.3395 vs. 0.3507, *p* = 0.0348). A trend of reduced mean degree of the whole brain was found in ALS patients (106.15 vs. 111.46, *p* = 0.0523).

**TABLE 2:** Global network analysis.

**Graph metrics**	**ALS patients**	**Healthy controls**	***P*-value**
Eglob	0.3395	0.3507	0.0348
Degree mean ± SD	106.15 ± 29.77	111.46 ± 29.10	0.0523
Eloc mean ± SD	0.3775 ± 0.0249	0.3854 ± 0.0244	0.12
BC mean ± SD	169.80 ± 245.61	170.06 ± 209.02	0.98

*Statistical significance was set at *p* < 0.05. Eglob, global efficiency; Eloc, local efficiency; BC, betweenness centrality.*

### Nodal Analysis

**FIGURE 2:**
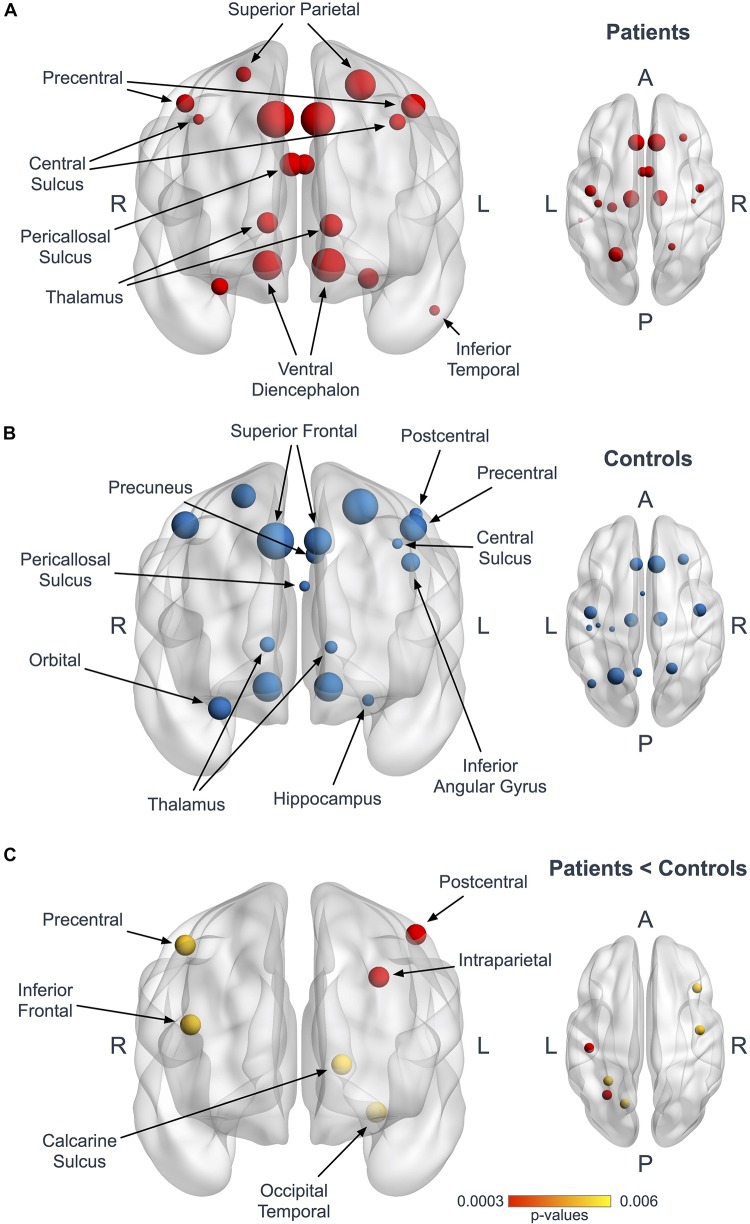
Hubs in ALS patients and controls. Panels **(A,B)** represent coronal and axial views illustrating the hubs in patients (**A**, red) and controls (**B**, blue). The size of the nodes is proportional to their degree values. Panel **(C)** shows the 6 nodes with significantly reduced mean degree in patients at the Bonferroni corrected threshold (red, p < 0.0003) and at the 1/n exploratory threshold (yellow, *p* < 0.006).

Using the Bonferroni corrected threshold, we found a significant decrease in ALS patients for the left postcentral gyrus (*p* < 0.0001) and for the left interparietal and transverse parietal sulcus (*p* < 0.0001) mean degree. No significant differences were identified in Eloc or BC in any nodes at this threshold. Furthermore, using the exploratory threshold, we found a significant decrease in the mean degree of the left calcarine sulcus (*p* = 0.0021), the left occipital temporal medial and lingual sulcus (*p* = 0.0009), the right precentral gyrus (*p* = 0.0021) and the right frontal inferior sulcus (*p* = 0.0009) (Figure 2C and Table 3).

**TABLE 3:** Significant mean degree nodes differences between ALS patients and controls.

**Nodes**	**ALS patients**	**Healthy controls**	***P*-value *^*a,b*^***
Left postcentral gyrus	133.64 ± 9.30	142.35 ± 8.16	0.0001^a^
Left interparietal and transverse parietal sulcus	126.68 ± 12.93	138.81 ± 7.93	0.0001^a^
Right precentral gyrus	139.52 ± 9.88	147.31 ± 6.83	0.0021^b^
Right frontal inferior sulcus	98.04 ± 15.00	110.69 ± 11.94	0.0009^b^
Left calcarine sulcus	76.52 ± 19.76	96.69 ± 21.03	0.0021^b^
Left occipital temporal medial and lingual sulcus	97.36 ± 12.13	109.11 ± 13.15	0.0009^b^

*Values are reported as mean ± SD. ^*a*^Statistical significance was set using Bonferroni corrections based on 164 brain regions (*p* < 0.0003). ^*b*^Statistical significance was set using an exploratory threshold 1/n (*p* < 0.006).*

### Hub Analysis

**FIGURE 3:**
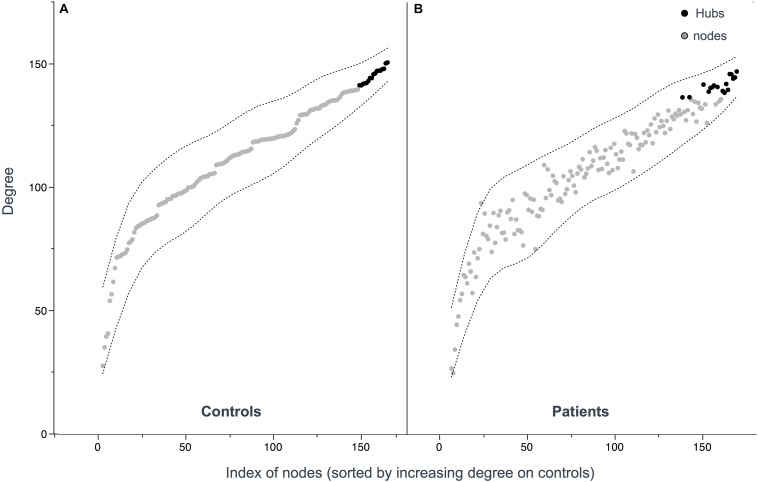
Hub distribution patterns among controls **(A)** and patients **(B)**. Hubs were defined as nodes with a degree one standard deviation higher than the average node degree. 17 hubs were identified in patients and 17 hubs in controls. Nodes **(A,B)** are sorted out by increasing values of degree in controls showing similar distribution.

**FIGURE 4:**
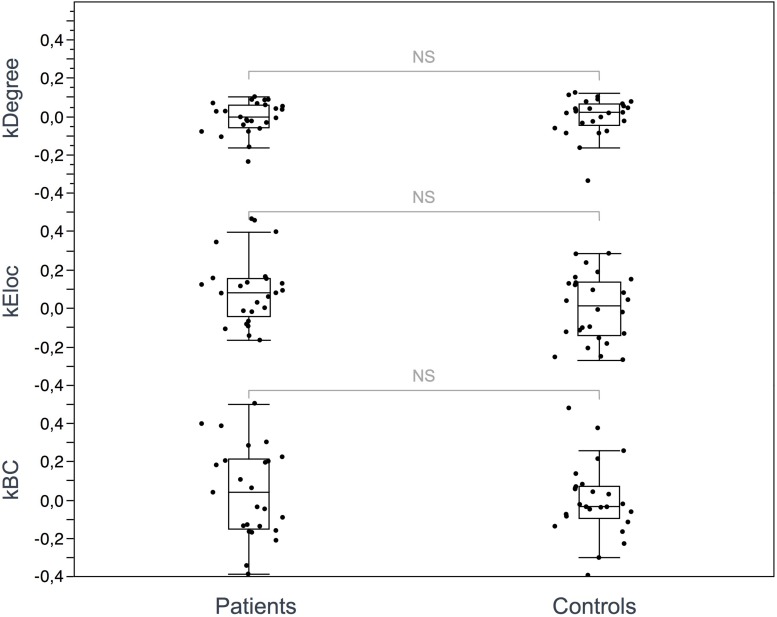
Hub disruption index. The box plots show the κ index of each subject for 3 metrics: κ-degree, κ-Eloc, κ-BC. No significant differences were identified between ALS patients and controls. Differences were considered significant at *p* < 0.05 for each metric.

Seventeen regions among 164 were defined as hubs both in patients and controls. The results are illustrated in Figure 2. Three hubs in controls were not identified as hubs in ALS patients: the left precuneus, the left parietal inferior angular gyrus and the left postcentral gyrus (Figure 3), but regarding the hub disruption index, no significant differences were found in κ-Eloc (*p* = 0.1270), κ-BC (*p* = 0.6511) or κ-degree (*p* = 0.6647) (Figure 4).

### Network-Based Statistics

**FIGURE 5:**
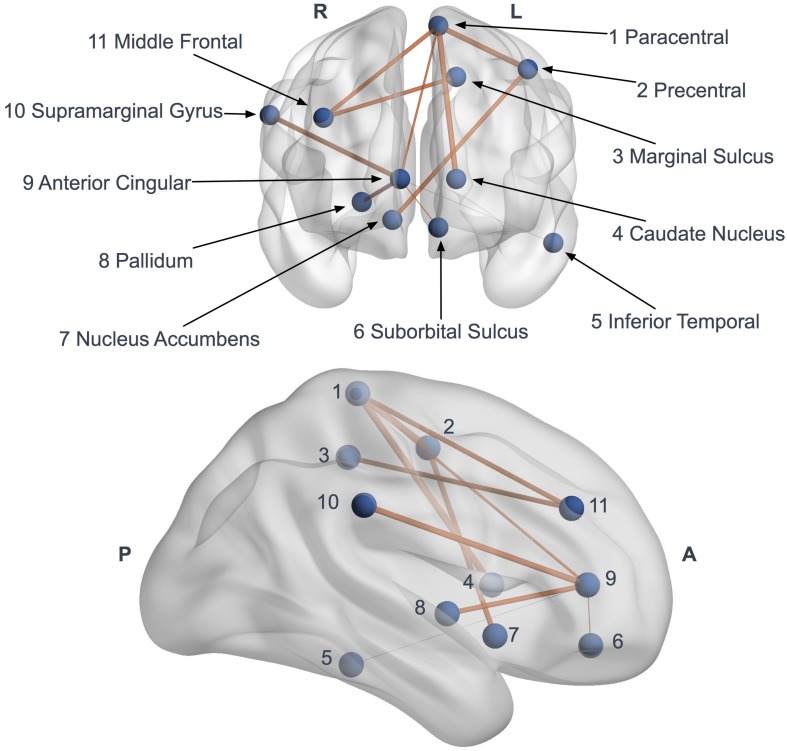
Network based statistics (NBS) impaired subnetwork. Coronal and sagittal views of impaired subnetwork nodes and interconnections identified by NBS (11 nodes and 10 edges). Edge diameter is based on the FA reduction (the larger connections are the more affected, with significant FA reduction).

Network-based statistics highlighted a subnetwork (11 nodes, 10 edges) of impaired connectivity in the ALS group (*p* = 0.015) (Figure 5). This network included the left precentral gyrus, the left paracentral gyrus, the left caudate nucleus, the left suborbital sulcus, the left inferior temporal sulcus, the left cingulate marginal gyrus, the right middle frontal gyrus, the right pallidum, the right accumbens area, the right anterior cingulate gyrus and sulcus and the right inferior superior parietal gyrus. The total FA sum of the edges in this subnetwork (NBS FA sum) was significantly reduced in ALS compared to controls (*p* < 0.0001).

### Correlation Between Connectivity Metrics and Clinical Parameters

Among the six nodes identified in the Nodal Analysis results section, degree in the right frontal inferior sulcus was correlated with the disease progression rate (*p* = 0.0089, rho = −0.5396) and a trend of association was also identified with ALSFRS-R scores (*p* = 0.0456, rho = 0.4033).

No significant correlations were found between the other nodes and ALSFRS-R score or disease progression rate. There were no significant associations between Eglob and ALSFRS-R score or disease progression rate.

## Discussion

This study provides evidence of disease-specific structural connectivity changes in ALS. We report global efficiency reductions in structural networks in ALS characterized by a significant decrease in Eglob and a trend of mean degree reduction. The use of the graph theory enables a topological study of the entire cerebral network, without targeting *a priori* regions of interest. Our results are in line with the reports of decreased of Eglob in previous studies (Zhang et al., 2019). Our findings also support the notion of widespread, multisystem, multi-network degeneration in ALS which has been conceived based on other structural (Keil et al., 2012; Menke et al., 2012; Bede et al., 2016; Müller et al., 2016) and functional methods (Agosta et al., 2011; Douaud et al., 2011; Geevasinga et al., 2017; Dukic et al., 2019; Nasseroleslami et al., 2019). Reduced global connectivity underpins the impaired integration of multiple cerebral circuits in ALS, which may be driven by long-range connectivity changes (He et al., 2009). Previous structural connectivity studies did not capture significant reductions in global parameters (Verstraete et al., 2011; Buchanan et al., 2015) which may be explained by differences in post processing pipelines which were used to generate structural connectomes (deterministic fiber tracking algorithm, number of tracts or different parcellation atlas).

At a local scale, we evaluated parcellated brain regions with multiple metrics (Eloc, degree and betweenness centrality) to determine if the global disturbances were due to the alteration of specific nodes. Nodal analysis demonstrated a significant reduction of degree in 6 out of 164 regions in ALS patients. These six nodes are located in pathognomonic brain regions closely associated with ALS. Precentral and frontal structural degeneration is a hallmark feature of ALS confirmed by a multitude of cortical gray and subcortical white matter studies (Kasper et al., 2014; Schuster et al., 2016; Mazón et al., 2018). The intraparietal sulcus is part of the supplementary motor areas which is also affected in ALS based on both functional and structural studies (Abidi et al., 2019). The involvement of the temporal lingual sulcus may be the substrate of the spectrum of cognitive changes observed in ALS (Abrahams et al., 2004; Phukan et al., 2007). Intrahemispheric connectivity alterations and the degeneration of the sensorimotor network have also been previously reported in ALS by studies demonstrating widespread pre- and postcentral FA reductions (Rose et al., 2012).

To explore internodal interactions, we used NBS (Zalesky et al., 2010). This tool permits the integrated analysis of a subgroup of nodes that belongs to a single altered network. In our study, we identified an impaired subnetwork centered on motor nodes: precentral, paracentral and frontal gyri. This subnetwork is comparable to the ones described in previous studies (Verstraete et al., 2011; Buchanan et al., 2015). Our finding of parietal and temporal lobe involvement is in line with recently proposed pathological staging systems (Brettschneider et al., 2013), and longitudinal connectivity studies (Verstraete et al., 2014).

Interestingly, no major topological reorganization of hubs was evidenced in ALS in the present study. The human connectome is known to follow characteristic topological patterns based on hubs, defined by a subnetwork of highly connected nodes with a high number of tracts playing a central role in the brain’s structural architecture. Due to their anatomical position and their high metabolic demands, these regions are considered particularly vulnerable in many neurological and psychiatric disorders (Buckner et al., 2009; Sharma et al., 2011; Rubinov and Bullmore, 2013; Gollo et al., 2018). Previous studies have shown that the selective hub degeneration occurs in a disease-specific pattern in several neurological and psychiatric conditions such as Alzheimer’s disease or schizophrenia (Crossley et al., 2014). Hub pathology in ALS, however, has not been specifically investigated to date and no graph theory based structural studies have been performed to evaluate the distribution of hubs and the extent to which they are reorganized in ALS patients relative to controls. We showed here that both the number and the distribution of hubs are similar between patients and controls. To our knowledge, this first use of the hub disruption index in ALS demonstrating the absence of hub reorganization, suggests that disease propagation in ALS does not follow hub-based patterns. Despite motor network changes evidenced by the NBS analysis, the precentral nodes kept their hub properties. Impairment of the global efficiency seems to be related to local structural abnormalities rather than a complex compensatory hub reorganization as reported in other disorders such as multiple sclerosis and epilepsy (Ridley et al., 2015; Faivre et al., 2016; Tur et al., 2018).

Noteworthy, we found associations between structural connectivity metrics and clinical parameters, such as disease progression rate and ALSFRS-R score. Correlations between brain imaging metrics and clinical parameters are often difficult to establish in ALS (Chipika et al., 2019) because motor disability is not merely due to upper but also lower motor neuron degeneration and the considerable clinical heterogeneity of the disease precludes direct clinico-radiological associations (Verstraete et al., 2015). It is therefore not surprising that no direct correlations were identified between clinical parameters and global connectivity measures. Node degree and their white matter integrity indexes seem to better explain the clinical deficits observed in ALS.

This study is not without limitations. The sample size of our ALS cohort is relatively limited which did not allow the phenotypic stratification of ALS patients into subgroups. Nonetheless, our study presents compelling evidence that graph-analyses and connectomics provide meaningful non-invasive insights into the degenerative changes of ALS. Furthermore, genetic information was not available for all patients which is a limitation as certain ALS genotypes may be associated with specific white matter alterations and more extensive network impairment (Menke et al., 2016; Floeter and Gendron, 2018). The lack of standardized cognitive evaluation is another limitation given the evidence of phenotype-specific morphometric changes along the ALS-FTD continuum (Omer et al., 2017). Future studies should therefore include ALS-FTD patients and ALS patients with behavioral and executive dysfunction to characterize the connectivity signature of these cognitive phenotypes. An additional limitation is the significantly higher proportion of male ALS patients in the present study. Given the evidence of gender-associated developmental and radiological features in ALS and healthy populations (Menzler et al., 2011; Vivekananda et al., 2011; Bede et al., 2014), future connectivity studies should seek to recruit gender-balanced cohorts.

## Conclusion

In conclusion, our structural connectivity study highlights a diffuse, non-focal network impairment in ALS without selective hubs vulnerability or topological network reorganization. These results suggest that clinical manifestations in ALS could be more driven by tract degeneration than by hub topology alterations. Based on these observations, a prospective longitudinal study is required to characterize dynamic progressive connectivity changes. Recent imaging studies have shown evidence of considerable presymptomatic structural changes in ALS-associated mutation carriers (Schuster et al., 2015; Querin et al., 2019). A connectomic analysis of such cohorts could identify early network alterations in the most vulnerable circuits and provide invaluable insights on disease propagation.

## Data Availability Statement

Anonymized datasets generated in this study may be available on request from the corresponding author, but are subject to institutional and EU data handling procedures.

## Ethics Statement

The studies involving human participants were reviewed and approved by Sud Mediterranee I Ethics Committee. The patients/participants provided their written informed consent to participate in this study.

## Author Contributions

All authors contributed scientifically to this manuscript. Clinical evaluations were performed by A-MG, AV, and SA. The methodological design was optimized by A-MG, MG, J-PR, and WZ. Image processing was undertaken by EF, AL, and BR. Data analyses were conducted by EF, A-MG, BR, J-PR, and WZ. The manuscript was drafted by EF, A-MG, and WZ. All co-authors have reviewed the manuscript for intellectual content.

## Conflict of Interest

The authors declare that the research was conducted in the absence of any commercial or financial relationships that could be construed as a potential conflict of interest.

## Footnotes

1 http://surfer.nmr.mgh.harvard.edu

2 https://www.fil.ion.ucl.ac.uk/spm/software/spm12/

3 https://fsl.fmrib.ox.ac.uk

4 http://www.brain.org.au/software/

## Abbreviations

ALS: amyotrophic lateral sclerosis
ALSFRS-R: revised ALS functional rating scale
BC: betweenness centrality
CSF: cerebrospinal fluid
DTI: diffusion tensor imaging
Eglob: global efficiency
Eloc: local efficiency
FA: fractional anisotropy
FTD: frontotemporal dementia
NBS: network-based statistics
ROI: region of interest.
